# Characterisation of tumour microvessel density during progression of high-grade serous ovarian cancer: clinico-pathological impact (an OCTIPS Consortium study).

**DOI:** 10.1038/s41416-018-0157-z

**Published:** 2018-06-29

**Authors:** Ilary Ruscito, Dan Cacsire Castillo-Tong, Ignace Vergote, Iulia Ignat, Mandy Stanske, Adriaan Vanderstichele, Jacek Glajzer, Hagen Kulbe, Fabian Trillsch, Alexander Mustea, Caroline Kreuzinger, Pierluigi Benedetti Panici, Charlie Gourley, Hani Gabra, Marianna Nuti, Eliane T. Taube, Mirjana Kessler, Jalid Sehouli, Silvia Darb-Esfahani, Elena Ioana Braicu

**Affiliations:** 1 Department of Gynecology, European Competence Center for Ovarian Cancer, Campus Virchow Klinikum, Charité – Universitätsmedizin Berlin, Corporate member of Freie Universität Berlin, Humboldt-Universität zu Berlin, Berlin Institute of Health, Berlin, Germany; 2 Cell Therapy Unit and Laboratory of Tumor Immunology, Department of Experimental Medicine, Sapienza University of Rome, Rome, Italy; 3 Translational Gynecology Group, Department of Obstetrics and Gynecology, Comprehensive Cancer Center, Medical University of Vienna, Waehringer Guertel 18–20, A-1090 Vienna, Austria; 4 Division of Gynaecological Oncology, Leuven Cancer Institute, Department of Gynaecology and Obstetrics, University Hospital Leuven, Catholic University of Leuven, Herestraat 49, B-3000 Leuven, Belgium; 5 Institute of Pathology, Charité – Universitätsmedizin Berlin, corporate member of Freie Universität Berlin, Humboldt-Universität zu Berlin, Berlin Institute of Health, Berlin, Germany; 6 Department of Obstetrics and Gynecology, University Hospital, LMU Munich, Marchioninistrasse 15, Munich, Germany; 7 Department of Gynecology and Gynecologic Oncology, University Medical Center Hamburg-Eppendorf, Martinistr. 46, Hamburg, Germany; 8 Department of Gynecology and Obstetrics, University Medicine of Greifswald, Greifswald, Germany; 9 Department of Gynecology, Obstetrics and Urology, Sapienza University of Rome, Rome, Italy; 10 Nicola Murray Centre for Ovarian Cancer Research, MRC IGMM, Western General Hospital, University of Edinburgh Cancer Research, UK Centre, Crewe Road South, Edinburgh, EH4 2XR UK; 11 Ovarian Cancer Action Research Centre, Department of Surgery and Cancer, Imperial College London, London, UK; 12 Clinical Discovery Unit, AstraZeneca, Cambridge, UK; 13 Department of Molecular Biology, Max Planck Institute for Infection Biology, Berlin, Germany

**Keywords:** Ovarian cancer, Molecular medicine

## Abstract

**Background:**

High-grade serous ovarian cancer (HGSOC) intratumoural vasculature evolution remains unknown. The study investigated changes in tumour microvessel density (MVD) in a large cohort of paired primary and recurrent HGSOC tissue samples and its impact on patients’ clinico-pathological outcome.

**Methods:**

A total of 222 primary (pOC) and recurrent (rOC) intra-patient paired HGSOC were assessed for immunohistochemical expression of angiogenesis-associated biomarkers (CD31, to evaluate MVD, and VEGF-A). Expression profiles were compared between pOCs and rOCs and correlated with patients' data.

**Results:**

High intratumoural MVD and VEGF-A expression were observed in 75.7% (84/111) and 20.7% (23/111) pOCs, respectively. MVD^high^ and VEGF^(+)^ samples were detected in 51.4% (57/111) and 20.7% (23/111) rOCs, respectively. MVD^high^/VEGF^(+)^ co-expression was found in 19.8% (22/111) and 8.1% (9/111) of pOCs and rOCs, respectively (*p* = 0.02). Pairwise analysis showed no significant change in MVD (*p* = 0.935) and VEGF-A (*p* = 0.121) levels from pOCs to rOCs. MVD^high^ pOCs were associated with higher CD3^(+)^ (*p* = 0.029) and CD8^(+)^ (*p* = 0.013) intratumoural effector TILs, while VEGF^(+)^ samples were most frequently encountered among BRCA-mutated tumours (*p* = 0.019). Multivariate analysis showed VEGF and MVD were not independent prognostic factors for OS.

**Conclusions:**

HGSOC intratumoural vasculature did not undergo significant changes during disease progression. High concentration of CD31^(+)^ vessels seems to promote recruitment of effector TILs. The study also provides preliminary evidence of the correlation between VEGF-positivity and BRCA status.

## INTRODUCTION

High-grade serous ovarian carcinoma (HGSOC) still accounts for the highest mortality rate among all ovarian cancer (OC) histotypes, with almost 80% of all new deaths from OC being caused by this distinct subgroup of ovarian tumours.^1–4^ International groups of opinion leaders have recognised the designing of new translational studies on recurrent and end-stage HGS tumour tissue samples as a key 'unmet need' in the understanding of HGSOC biology and clonal evolution.^4^

In this scenario, analysis of the evolution process affecting intratumoural vasculature during HGSOC progression is a pivotal issue to be still elucidated.

After decades of paralysis in primary OC first-line chemotherapy treatment, indeed, incorporation of bevacizumab in the upfront regimen for advanced newly diagnosed disease^5^ has changed the 'standard of care paradigm' of advanced primary OC, although characterised by less survival impact than expected.^6–8^ Thus, understanding changes in the vasculature or identification of prognostic biomarkers of response to vasculature targeting is needed. Unfortunately, there are currently no predictive biomarkers to tailor bevacizumab treatment in OC patients.

A full knowledge of molecular changes involving intratumoural vasculature from primary to recurrent HGSOC is still lacking and may provide new opportunities to: (1) tailor treatment with currently available anti-angiogenetic agents, (2) shed light on acquired resistance mechanisms, and (3) develop new targeted therapies.

The aim of this study was to identify changes occurring from primary to recurrent HGSOC in tumour tissue expression of the angiogenesis-associated biomarkers CD31, applied for detecting microvessels density (MVD),^9–11^ and VEGF-A,^12^ by analysing a large cohort of paired primary and recurrent HGSOC tissue samples. Secondary endpoints included the correlation of biomarkers expression with patients’ clinico-pathological characteristics and survival data.

## Materials and methods

### Sample Collection

Paired cancer tissue samples belonging to HGSOC patients were collected during primary and secondary cytoreduction. Patients were treated with primary debulking surgery followed by platinum-based chemotherapy between 1985 and 2013, and were retrospectively and consecutively selected from OCTIPS (Ovarian Cancer Therapy–Innovative Models Prolong Survival, Agreement No.279113-2) Consortium database. Included patients underwent both primary (pOC) and recurrent (rOC) surgery in one of the European Gynaecologic Oncology referral Centers of the following Institutions: Charité Universitätsmedizin Berlin, Germany; Catholic University of Leuven, Belgium; Imperial College, London, UK; University of Edinburgh, UK; University Medical Center Hamburg-Eppendorf, Germany.

Inclusion criteria were: availability of paired primary and recurrent cancer tissue samples from HGSOC patient together with clinical annotation. Exclusion criterion was: neoadjuvant chemotherapy treatment, due to the need to analyse primary chemo-naïve tumours. Approval from each local ethics committee was obtained (EK207/2003, ML2524, 05/Q0406/178, EK130113, 06/S1101/16). All included samples underwent central histopathological assessment to confirm HGSOC histology and ensure tumour tissue content and quality.

### Immunohistochemistry

Tissue microarrays (TMA) were constructed for immunohistochemical staining. Each primary and recurrent tumour tissue sample was represented within the TMA by two tumour cores, each containing at least 90% of cancer cells.

Sections from TMA were deparaffinised in xylol, rehydrated in graded alcohol, and boiled in pressure cooker for 5 minutes in citrate buffer (pH = 6), for CD31 staining, or in EDTA (pH = 9), for VEGF staining. Rabbit anti-human CD31 antibody (clone ab32457; Abcam, Cambridge, MA, USA) and rabbit anti-human VEGF-A antibody (clone A-20; Santa Cruz Biotechnology, Dallas, TX, USA) were diluted 1:20 and 1:250, respectively, and incubated on slides for 60 minutes at room temperature. Bound antibodies were visualised using DAKO Real Detection System and DAB + (3,3′-diaminobenzidine; DAKO, Glostrup,Denmark) as a chromogen. Finally, the slides were co-stained with hematoxylin.

CD31 stained samples were assessed in terms of MVD. MVD was determined by averaging the number of vessels from three distinct areas of tumour with highest vessels density examined at ×200 magnification.^13–15^

Samples were further classified into 'MVD^high^' (≥16.3 vessels) or 'MVD^low^' (<16.3 vessels), establishing the cut-off level of MVD count for dichotomisation at first quartile (primary samples), being the value able to maximise difference in OS hazard ratio^13,15,16^ (Table S1).

For VEGF staining evaluation, the number of stained tumour cells within the whole TMA cores (0% = 0; 1–10% = 1; 11–50% = 2; >50% = 3) was multiplied with the intensity of staining (negative = 0; weak = 1; moderate = 2; strong = 3),^17^ resulting in a semiquantitive immunoreactivity score (IRS) ranging from 0 to 9. Samples were classified as 'VEGF^(+)^', for VEGF-high tumour expression (IRS = 4–9), or as 'VEGF^(−)^', for absent/weak focal staining (IRS = 0–3).

As positive control for IHC were used human liver sections. Samples staining was assessed independently by two co-authors (IR and SDE).

### Patients’ clinico-pathological data

Patients’ clinico-pathological data, including somatic-BRCA status from 52 included patients, were retrieved from OCTIPS Consortium database.^18^ GCIG criteria were applied to define platinum-resistance and platinum-sensitivity.^19^ RECIST Criteria were applied during patients’ follow-up to define HGSOC relapse.^20^ No residual tumour was defined intraoperatively by the surgeon in case no macroscopic tumour could be detected at the end of cytoreduction.

In order to investigate any association between different tumour vasculature profiles and intratumoural immune infiltrate in both pOCs and rOCs, MVD and/or VEGF profiles were matched with previous OCTIPS data on tumour infiltrating lymphocytes (TILs), assessed through the immunohistochemical expression of CD3, CD4, and CD8 biomarkers, as previously reported.^21^ Furthermore, immunosuppressive TILs were evaluated through the expression of T-regulatory cells-specific biomarker FoxP3, using the mouse anti-human FOXP3 antibody (clone ab20034; Abcam, Cambridge, MA, USA, 1:200, 1.5 h at room temperature). The count of stained FoxP3-positive TILs was then performed automatically with the *VM Scope Quantifier*, as previously reported.^21^

### Statistical Analysis

Statistical analysis was performed using SPSS version 22.0 (SPSS Inc,Chicago,IL,USA). Difference in biomarker expression between pOCs and rOCs was assessed through the correlation test (Spearman coefficient, 2-tailed) and 'Wilcoxon signed rank' non-parametric test for related samples. Fisher’s exact test was applied to correlate MVD and/or VEGF tumour expression with patients’ clinico-pathological categorical data. Patients’ progression-free interval (PFI), progression-free survival (PFS), and overall survival (OS) were identified through Kaplan–Meier analysis (Log-Rank test). PFI was defined as the time interval from the last adjuvant chemotherapy to relapse, whereas progression-free survival (PFS) was established as the time interval between first recurrence diagnosis and tumour progression. Univariate and multivariate survival analyses were performed applying Cox-regression model. Multivariable models were obtained among variables reporting a *p*-value ≤ 0.1 in univariate analysis. *p*-values ≤ 0.05 were evaluated statistically significant.

## Results

A total of 222 intra-patient paired primary and recurrent HGSOC tissue samples derived from 111 patients were included. Patients’ characteristics are listed in Table 1. To note, only 2/111 (1.8%) patients received bevacizumab in front-line chemotherapy, thus the staining of recurrent samples have not been influenced by first-line administration of anti-angiogenetic compounds.

**Table 1:** Patient characteristics

Patients *n*	111
Age	
Median (range)	56 y (33y-77y)
FIGO Stage (%)	
­ I	2 (1.8%)
­ II	5 (4.5%)
­ III	93 (83.8%)
­ IV	11 (9.9%)
Residual tumour after PDS:	
­ No Residual Tumour	89 (80.2%)
­ Any Residual Tumour	22 (19.8%)
Type of first-line CHT	
With bevacizumab	2 (1.8%)
­ Without bevacizumab	109 (98.2%)
Type of second-line CHT	
With bevacizumab	8 (7.2%)
­ Without bevacizumab	103 (92.8%)
Platinum response after primary treatment	
­ Platinum sensitive	90 (81.1%)
­ Platinum resistant	18 (16.2%)
­ Unknown	3 (2.7%)
Platinum response after treatment for disease relapse	
­ Platinum sensitive	59 (53.2%)
­ Platinum resistant	12 (10.8%)
­ Missing	40 (36%)
Somatic-BRCA status	
­ BRCA wt	31 (27.9%)
­ BRCA 1/2 mutation	21 (18.9%)
­ Unknown	59 (53.2%)
Maximum follow-up time	214 months
Median OS	63 months

*CHT* Chemotherapy, *OS* Overall survival, *PDS* Primary debulking surgery, *wt* wild type

### MVD staining

**Fig. 1:**
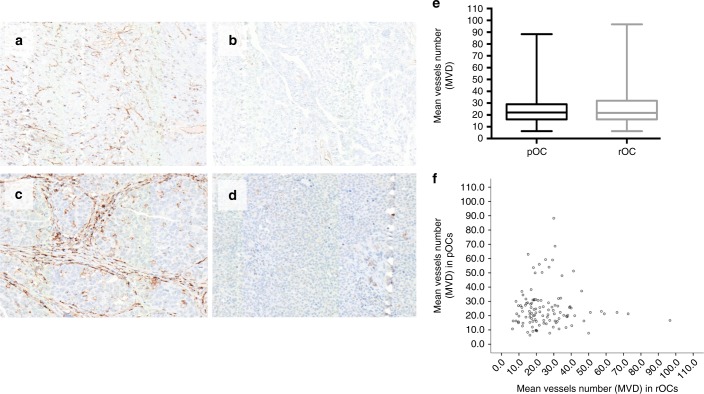
CD31 immunohistochemistry staining for intratumoural MVD assessment: MVD^high^ (**a**) and MVD^low^ (**b**) pOC samples; MVD^high^ (**c**) and MVD^low^ (**d**) rOC samples. ×400 magnification; MVD count among primary and recurrent tumours (box plot (**e**) and scatter plot (**f**))

MVD^high^ staining was detected in 75.7% (84/111) of pOC and in 51.4% (57/111) of rOC, whereas MVD^low^ staining was found in 24.3% (27/111) and in 48.6% (54/111) of pOC and rOC, respectively. MVD^low^ staining was twice as prevalent in relapsed tumours compared to primary disease (*p* = 0.0003, Fisher’s exact test, Fig. 1a–d). Nevertheless, globally, pairwise analysis revealed no tendency towards a change in MVD to higher or lower levels in recurrent samples (*p* = 0.935, Wilcoxon test; Fig. 1e), as well as no significant correlation between pOCs and rOCs in MVD was reported (Spearman correlation, *p* = 0.920; Spearman coefficient: 0.01).

### VEGF-A expression

**Fig. 2:**
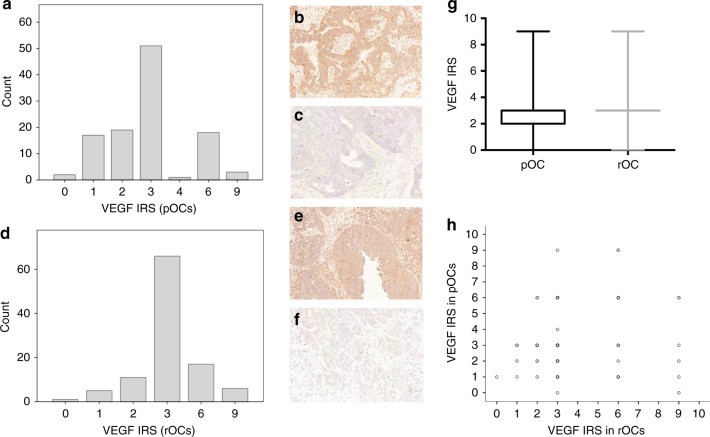
VEGF-A immunohistochemistry staining. VEGF-A IRS distribution in primary (**a**) and recurrent (**d**) tumour samples. pOCs, VEGF^(+)^ (**b**) and VEGF^(−)^ (**c**); rOCs, VEGF^(+)^ (**e**) and VEGF^(−)^ (**f**); VEGF-A IRS among primary and recurrent tumours (box plot (**g**) and scatter plot (**h**))

VEGF IRS distribution in both pOCs and rOCs is shown in Fig. 2a, d. The same percentage of VEGF^(+)^ (20.7%, 23/111) and VEGF^(−)^ (79.3%, 88/111) tumour samples was found between pOCs and rOCs, respectively, (*p* = 1, Fisher’s exact test, Fig. 2b, c, e, f), although no significant correlation between pOCs and rOCs VEGF IRS values could be observed (*p* = 0.505, Spearman coefficient 0.06). Furthermore, pairwise analysis confirmed no tendency towards a change in VEGF IRS levels at tumour relapse (*p* = 0.121, Wilcoxon test; Fig. 2g).

### MVD^high^ and VEGF^(+)^ co-expression in pOCs vs rOCs.

MVD^high^ and VEGF^(+)^ co-expression was more frequent in pOCs group (22/111, 19.8%) compared to rOCs (9/111, 8.1%) (*p* = 0.02, Fisher’s exact test, Fig. S1).

### Relationship between MVD and/or VEGF-A expression with TILs.

Results showed that MVD^high^ levels in pOCs samples were associated with higher CD3^(+)^ (*p* = 0.029, Mann–Whitney test) and CD8^(+)^ (*p* = 0.013) effector TILs, but not with a higher FoxP3^(+)^ (*p* = 0.443) T-regulatory cells infiltrate. To note, the correlation between MVD and CD3^(+)^/CD8^(+)^ TILs disappeared at tumour recurrence. No significance between pOCs or rOCs VEGF expression or MVD^high^ + VEGF^(+)^ co-staining with TILs was reported (Fig. S2, Table S2).

### MVD and/or VEGF-A profiles and patients’ clinico-pathological factors

Analysis on the correlation between MVD and/or VEGF expression in pOCs with patients’ clinico-pathological characteristics is shown in Table 2. In particular, VEGF^(+)^ primary HGSOCs and MVD^high^/VEGF^(+)^ primary samples were most frequently encountered among somatic-BRCA-mutated tumours compared to somatic-BRCA wild-type cases (*p* = 0.019, Fisher’s exact test). No further significant associations between different intratumoural vasculature profiles and patients’ age at diagnosis, FIGO stage, residual tumour after primary debulking or first-line platinum response was identified.

**Table 2:** Association of MVD and/or VEGF expression with patients’ clinico-pathological characteristics (pOCs)

Clinico-pathological factors	Total *N*	MVD (pOC)			VEGF (pOC)			MVD high + VEGF pos co-expression (pOC)		
		High	Low	*P*	High	Low	*P*	Yes	No	*P*
Patients’ Age										
<56 y	53	39	14	0.663	13	40	0.360	13	40	0.246
≥56 y	58	45	13		10	48		9	49	
FIGO Stage										
I/II	7	4	3	0.358	2	5	0.633	2	5	0.624
III/IV	104	80	24		21	83		20	84	
Residual tumour after first cytoreductive surgery										
No residual	89	67	22	1	18	71	0.775	17	72	0.767
Any residual	22	17	5		5	17		5	17	
Platinum-sensitivity status after primary treatment										
Platinum sensitive	90	71	19	0.133	18	72	0.530	17	73	0.521
Platinum resistant	18	11	7		5	13		5	13	
Somatic-BRCA status										
BRCA-WT	31	26	5	0.105	3	28	0.019	3	28	0.019
mBRCA1/2	21	13	8		8	13		8	13	

Decrease of VEGF expression in rOCs was observed only in BRCA-mutated patients (*p* = 0.053, Wilcoxon test), although this association did not reach statistical significance (Fig. S3).

### Survival

**Fig. 3:**
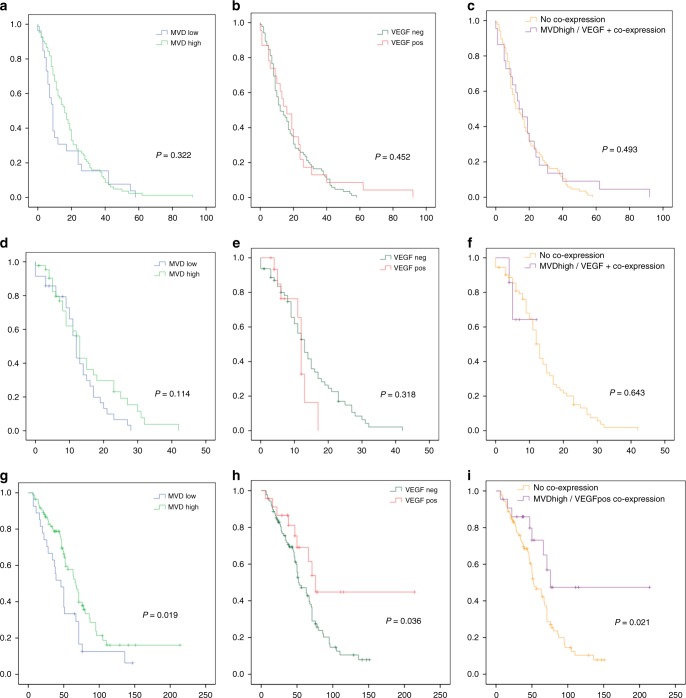
MVD and/or VEGF status and progression-free survival after primary (PFI (**a**), (**b**), (**c**)) and recurrent (PFS, (**d**), (**e**), (**f**)) disease. **g**–**i** MVD and/or VEGF status at primary disease and overall survival. '*x*-axis': months, '*y*-axis': survival probability

Patients, whose pOCs resulted MVD^high^, VEGF^(+)^ or co-stained for both biomarkers, were found to have a significantly improved OS compared to patients without these intratumoural profiles at primary disease (Fig. 3g–i). In particular, median OS for MVD^high^ and MVD^low^ patients was 67 and 46 months, respectively (*p* = 0.019), median OS for VEGF^(+)^ and VEGF^(−)^ patients resulted 76 vs 52 months, respectively (*p* = 0.036), while median OS for patients with co-stained pOCs was 76 months, compared to 52 months in women without co-expression (*p* = 0.021).

On the contrary, no influence of pOCs or rOCs MVD and/or VEGF expression on patients’ time to progression after primary (PFI) or first recurrent disease (PFS) was reported (Fig. 3a–f).

Multivariate analysis for OS and PFI was carried out on the whole patients’ population (*n* = 111) and also on the subgroup of patients (*n* = 52) with known tumour somatic-BRCA status. Table 3a, b shows that VEGF-A was not found to be an independent prognostic factor for OS anymore when considering also somatic-BRCA mutational status. Only somatic-BRCA mutation (HR: 0.354, CI 95%: 0.133–0.994; *p* = 0.038), high CD4^(+)^ TILs (HR: 0.997, CI 95%: 0.995–1.000; *p* = 0.038) and first-line platinum response (HR: 0.216, CI 95%: 0.051–0.991; *p* = 0.037) were found to independently improve HGSOC patients’ OS.

**Table 3:** Multivariate analysis for OS

	HR (95% CI)	*P*
a: Whole population (*n* = 111)		
Overall survival		
Age (≥56 y vs <56 y)	1.155 (0.683–1.953)	0.590
FIGO stage (III/IV vs I/II)	2.507 (0.621–10.127)	0.197
Residual tumour (any residual vs no residual)	1.610 (0.875–2.962)	0.126
MVD (high vs low)	0.818 (0.417–1.604)	0.558
VEGF (positive vs negative)	0.420 (0.178–0.991)	**0.048**
FoxP3 mean number	0.963 (0.778–1.191)	0.727
CD3 mean number	1.000 (0.998–1.002)	0.786
CD4 mean number	1.000 (0.999–1.001)	0.925
CD8 mean number	1.000 (0.998–1.002)	0.846
Platinum response (Plat. Sens. vs Plat. Resist)	0.229 (0.104–0.506)	**<** **0.001**
b: Only somatic-BRCA-tested population (*n* = 52)		
Overall survival		
Age (≥56 y vs <56 y)	1.017 (0.410–2.524)	0.971
FIGO stage (III/IV vs I/II)	1.506 (0.091–24.829)	0.775
Residual tumour (any residual vs no residual)	1.417 (0.259–7.755)	0.687
MVD (high vs low)	0.747 (0.243–2.291)	0.609
VEGF (positive vs negative)	0.440 (0.127–1.526)	0.196
FoxP3 mean number	0.683 (0.439–1.061)	0.090
CD3 mean number	0.998 (0.994–1.001)	0.132
CD4 mean number	0.997 (0.995–1.000)	**0.038**
CD8 mean number	0.998 (0.994–0.997)	0.438
Somatic-BRCA status (BRCA–mut vs BRCA wt)	0.354 (0.133–0.994)	**0.038**
Platinum response (Plat. Sens. vs Plat. Resist)	0.216 (0.051–0.991)	**0.037**
c: Whole population (*n* = 111)		
Progression-free interval		
Age (≥56 y vs <56 y)	1.067 (0.692–1.644)	0.770
FIGO stage (III/IV vs I/II)	2.447 (0.892–6.711)	0.082
Residual tumour (any residual vs no residual)	1.009 (0.568–1.794)	0.974
MVD (high vs low)	1.445 (0.832–2.511)	0.191
VEGF (positive vs negative)	0.945 (0.541–1.652)	0.843
FoxP3 mean number	0.984 (0.832–1.162)	0.845
CD3 mean number	1.000 (0.999–1.001)	0.835
CD4 mean number	1.000 (0.999–1.001)	0.698
CD8 mean number	1.000 (0.998–1.002)	0.845
d: Only somatic-BRCA-tested population (*n* = 52)		
Progression-free interval		
Age ( ≥ 56 y vs < 56 y)	1.121 (0.542–2.318)	0.759
FIGO stage (III/IV vs I/II)	18.261 (1.282–260.172)	**0.032**
Residual tumour (any residual vs no residual)	1.391 (0.280–6.918)	0.687
MVD (high vs low)	0.884 (0.375–2.081)	0.777
VEGF (positive vs negative)	0.916 (0.400–2.095)	0.834
FoxP3 mean number	0.868 (0.659–1.145)	0.317
CD3 mean number	0.998 (0.995–1.001)	0.159
CD4 mean number	0.996 (0.993–0.998)	**0.001**
CD8 mean number	0.999 (0.995–1.003)	0.719
Somatic-BRCA status (BRCA-mut vs BRCA wt)	0.982 (0.462–2.087)	0.962

Multivariate analysis for OS carried out on (a) the whole patients’ population (*n* = 111), (b) only somatic-BRCA-tested population (*n* = 52) and multivariate analysis for PFI carried out on (c) the whole patients’ population (*n* = 111), (d) only somatic-BRCA-tested population (*n* = 52). Bold values indicate significant *p* values (<0.05)

When analysing the PFI in patients with or without BRCA somatic mutations, advanced FIGO stage (HR: 18.261, CI 95%: 1.28–260.17; *p* = 0.032) and low CD4^(+)^ TILs (HR: 0.996, CI 95%: 0.993–0.998; *p* = 0.001) were the only independent poor prognostic factors (Table 3c, d).

## Discussion

In the last decade, 'omics' sciences provided fundamental insight into the understanding of HGSOC biology,^3^ showing as one distinct malignancy with its own characteristic phenotype, aetiology and progression profile.^22^ Although known for its aggressive behaviour, HGSOC has a higher change to show durable response after first-line chemotherapy, compared to other OC histologies,^23^ as well as its common platinum-sensitivity allows it to access a more varied panel of experimental second-line combinations.^24^ Unfortunately, progression from HGSOC is often rapid and chemo-resistance develops.^4^

In this context, understanding the biological changes occurring to HGSOC during disease progression is an essential issue through which new identified biomolecular signatures, marking the HGSOC clinical evolution, could help developing new tailored treatment strategies.

In this study, OCTIPS Consortium aimed to identify modifications involving HGSOC intratumoural vasculature from primary to recurrent disease, by assessing the evolution of cancer MVD and VEGF-A expression. Results showed that: (1) MVD and/or VEGF levels did not undergo significant changes from pOC to rOC (being in line with already available clinical findings, as bevacizumab is showing mild improvement in PFS, in both primary and relapsed situation);^5,7,8^ (2) High MVD levels in pOC seems to sustain the intratumoural recruitment of effector TILs and were associated with better OS in HGSOC patients; (3) VEGF^(+)^ HGSOCs were most frequently encountered among somatic-BRCA-mutated tumours and VEGF-positivity correlates with better OS in this HGSOC cohort; (4) MVD and VEGF were not independent prognostic factor for OS when taking into account the BRCA mutational status and TILs profile.

The definition of 'intratumoural microvessel density' has been coined in the middle of 90’s to objectivise the entity of blood supply available within the tumour mass to sustain cancer growth.^25^ Intratumoural vessels are usually characterised by impaired vascular maturation, poor functionality and defects in endothelial architecture. Immaturity of the new generated tumour-associated vasculature results in excessive permeability, poor perfusion and imperfect blood flow.^26^

During the last 20 years, different studies recognised 'high' MVD a poor prognostic factor for cancer patients,^27–29^ including women affected by OC.^30^ Different biomarkers have been adopted to assess MVD in OC, including Von Willebrand Factor, CD105, CD34 and CD31, being CD34 the most used MVD detector and the biomarker associated with the poorest HR for OS (HR: 1.67, CI 95%: 1.36–2.35) compared to other MVD detectors (HR: 1.32, CI 95%: 0.82–1.82).^30^

CD31, also known as 'platelet endothelial cell adhesion molecule-1' (PECAM-1) is a transmembrane glycoprotein expressed on endothelial cells, platelets, neutrophils and T-cells. It is a key factor to maintain the integrity of endothelial cells permeability barrier and to promote the controlled activation of T-cells and their survival,^11,31,32^ thus being expression of a normalised endothelium able to sustain the correct trafficking of T-cells into the tumour. In line with CD31 biological role, we observed that MVD^high^ levels in pOCs samples correlated with higher CD3^(+)^ and CD8^(+)^ TILs, but not with a higher FoxP3^(+)^ T-lymphocytes infiltrate, thus suggesting that a high concentration of intratumoural CD31^(+)^ vessels might be able to promote the intratumoural recruitment of effector T-cell populations, thus ultimately improving patients’ survival.^33^ Recently, Bais et al.^16^ identified CD31-dependent MVD as a predictive biomarker for bevacizumab response in first-line treated OC patients. This finding might be consequence of intratumoural endothelial maturity, represented by high CD31-dependent MVD levels, able to ensure a normalised blood flow, which is pivotal for intratumoural drug delivery and efficacy.^26^

Vascular Endothelial Growth Factor (VEGF) is a key angiogenetic cytokine that regulates cell mitosis and endothelial cells permeability.^34^ Overexpression of VEGF has been found to correlate with cancer relapse and decreased survival in patients affected by different solid tumours, including OC.^35^ Despite previous studies, absence of significant changes in MVD and VEGF profile following disease progression of this unique cohort, indicates that these markers are not major drivers of molecular cancer evolution in vivo, but rather remain supportive factors.

One of the most intriguing outcomes of our study is that VEGF-A overexpression in pOC has been most frequently found among patients with a cancer somatic mutation of BRCA1/2 genes. This finding is in line with two other previously published papers. In 2013, Danza^36^ observed that BRCA-mutated breast cancer patients reported higher levels of VEGF mRNA (*P* = 0.04) compared with those without BRCA mutations. In 2016, another study revealed that a VEGF-dependent gene signature (VDGs) was overexpressed in OC BRCA mutation carriers.^37^ An interesting hypothesis explaining the linking between BRCA1 mutation and VEGF overexpression in HGSOC has been recently proposed: in 2015 Desai A and Colleagues^38^ pointed out that wild-type BRCA1 binds to Ubc9, which induces Caveolin-1 expression, downregulates VEGF and regulates endothelial function in normal ovaries and fallopian tubes. In HGSOC with BRCA1 dysfunction, Ubc9 is not binded and this inhibits Caveolin-1 expression causing increased VEGF levels, loss of endothelial function and accumulation of ascites. Compared to these previous studies, we also confirmed in our cohort the positive influence of BRCA mutations on OC patients’ survival,^39,40^ as well as the significant association between BRCA mutation and VEGF-positivity determined VEGF-positivity a good prognostic factor in our HGSOC series. This result may also reflect the highly selection of the sample analysed, which only included HGSOC patients, who can also undergo secondary cytoreductive surgery for recurrence. These patients have usually good performance status and low tumour burden, so there is a selection of patients with a better clinical outcome.^41^ Furthermore, patients have been treated in high volume centres, with high experience in surgical treatment of ovarian cancer. Most Centers have been also approved and allowed to participate in the LION (ClinicalTrials.gov Identifier: NCT00712218), DESKTOP III (ClinicalTrials.gov Identifier: NCT01166737) and TRUST (ClinicalTrials.gov Identifier: NCT02828618) studies, based on the high quality of the tumour debulking.

Nevertheless, further studies aiming to assess the association between BRCA mutation and VEGF overexpression would provide new instrument to personalise treatment with anti-angiogenetic agents among BRCA-mutated and BRCA wild-type OC patients.^42^ In this scenario, the randomised phase III clinical trial ENGOT-ov25/PAOLA-1 (ClinicalTrials.gov Identifier: NCT02477644), which combines in advanced OC patients bevacizumab-based first-line treatment with or without the PARP-Inhibitor olaparib, could be able to add evidence concerning functional impact of VEGF expression in tumours with impaired homologous DNA repair mechanism.

To our knowledge, this is the first study analysing the changes occurring in intratumoural vasculature during disease progression in the largest cohort of paired primary and recurrent HGSOC samples. It firstly demonstrated that the vascular architecture within the tumour mass, in absence of anti-angiogenic agents administration, is maintained relatively stable during the natural course of the disease. Furthermore, the subanalysis on patients with known somatic-BRCA status increases the value of findings by taking into account the impact of BRCA status on patients’ survival^39,40^ and provides preliminary evidence of the correlation between VEGF-positivity and BRCA mutation.

The main limitation of the study is its retrospective nature. One of the strengths of this analysis is the large sample size of paired primary and recurrent tumour tissue samples belonging to the same cancer subtype (*n* = 222), the high quality of specimens and the systematisation of multicentric patients’ clinico-pathological data. Furthermore, inclusion of patients not subjected to the bevacizumab-based first-line chemotherapy, increase the reliability of the results in comparing intratumoural vasculature profiles from primary to recurrent disease.

Future study on a larger population with known BRCA status, who has been subjected to bevacizumab-based first-line chemotherapy, is warranted to clarify the role of MVD and VEGF in predicting bevacizumab response in both BRCA-wt and BRCA-mutated HGSOC patients.

## Electronic supplementary material

Figure S1 (supplementary)

Figure S2 (supplementary)

Figure S3 (supplementary)

Table S1 (supplementary)

Table S2 (Supplementary)

Supplementary Figure Captions

## Availability of data and materials

Data supporting the results reported are stored in the OCTIPS Consortium database. The documentation of clinical and patient’s data was managed with “AlcedisTRIAL the web based documentation system” of Alcedis GmbH, Winchesterstr. 3, 35394 Giessen, Germany.
