# Supplementary figures and images for: Characterisation of tumour microvessel density during progression of high-grade serous ovarian cancer: clinico-pathological impact (an OCTIPS Consortium study)
Source: Br J Cancer. 2018 Jun 29;119(3):330–8. doi: 10.1038/s41416-018-0157-z (PMC6070919; doi:10.1038/s41416-018-0157-z)

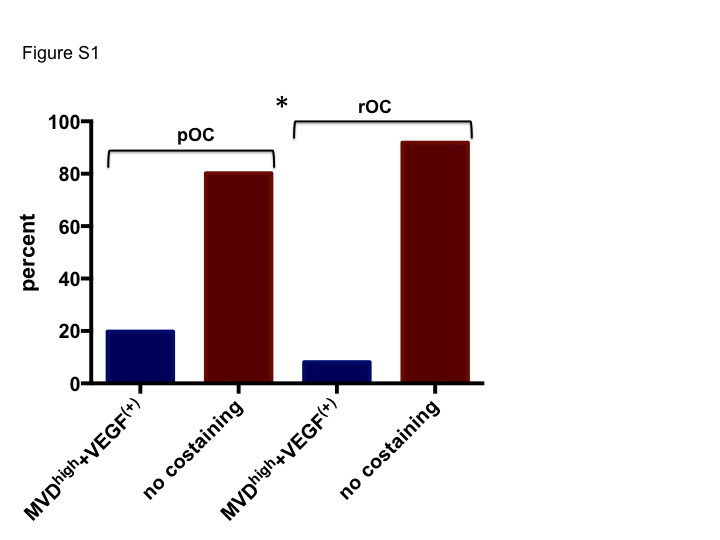

Supplement: Supplementary file 1 — Figure S1 (supplementary) [file 41416_2018_157_MOESM1_ESM.tif]

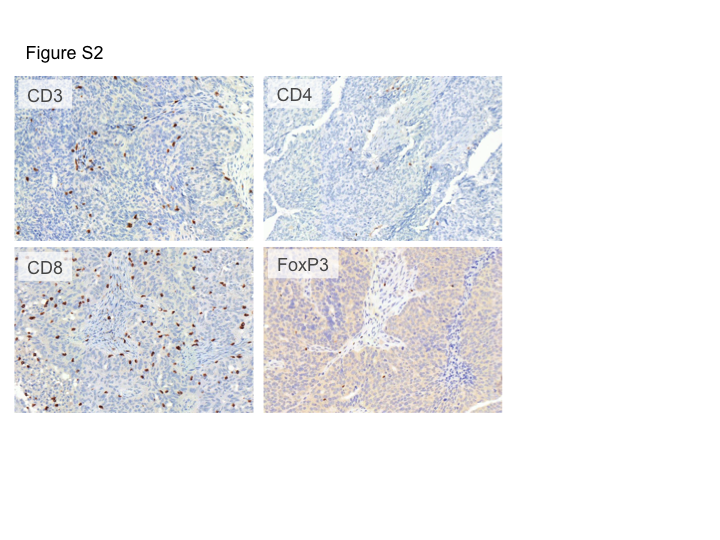

Supplement: Supplementary file 2 — Figure S2 (supplementary) [file 41416_2018_157_MOESM2_ESM.tif]

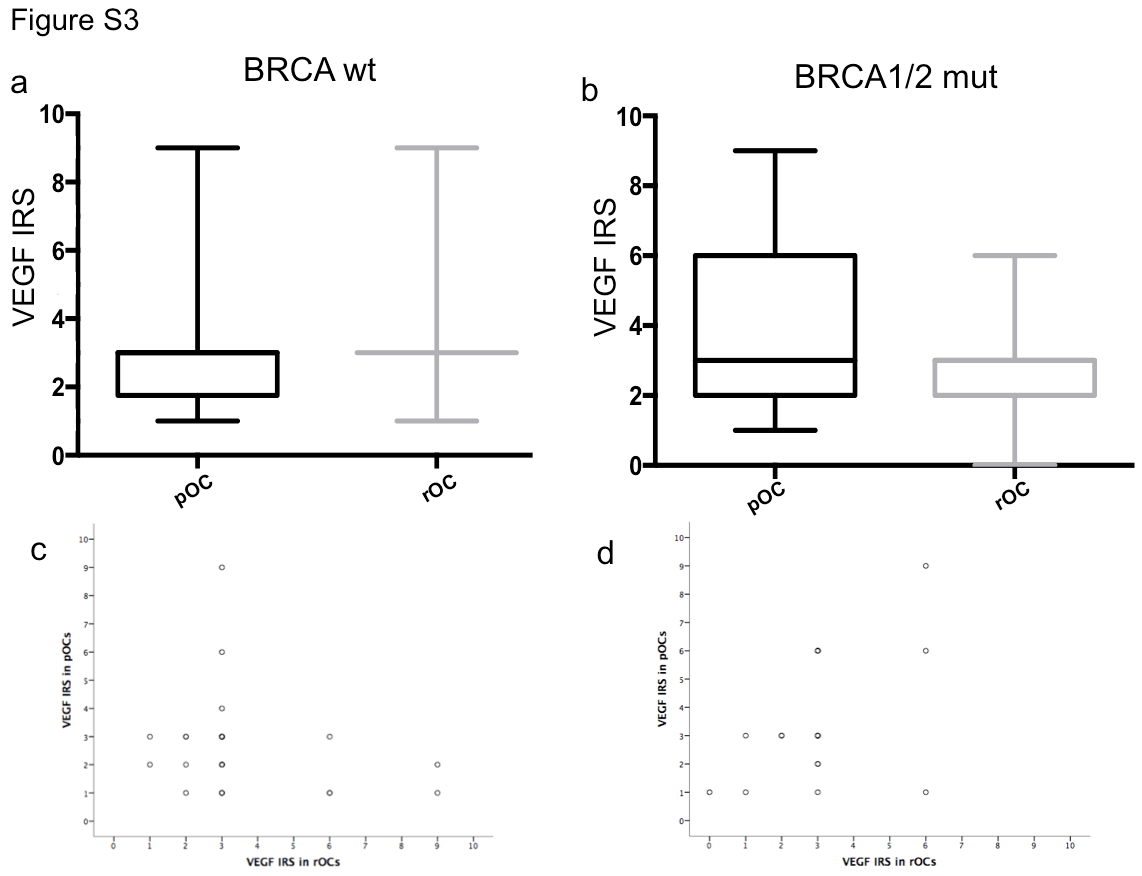

Supplement: Supplementary file 3 — Figure S3 (supplementary) [file 41416_2018_157_MOESM3_ESM.png]
