# Supplementary material for: Characterisation of tumour microvessel density during progression of high-grade serous ovarian cancer: clinico-pathological impact (an OCTIPS Consortium study)
Source: Br J Cancer. 2018 Jun 29;119(3):330–8. doi: 10.1038/s41416-018-0157-z (PMC6070919; doi:10.1038/s41416-018-0157-z)
Supplement: Supplementary file 4 — Table S1 (supplementary) [file 41416_2018_157_MOESM4_ESM.docx]

|  | No. death events/tot. No. patients | | Median OS (months) | | HR (C.I.95%) | P |
| --- | --- | --- | --- | --- | --- | --- |
| Cut-off | MVD^high^ | MVD^low^ | MVD^high^ | MVD^low^ |  |  |
| Q1 (first quartile) | 50/84 | 24/27 | 67 | 46 | 1.77 (1.08-2.88) | **0.023** |
| Q2 (median) | 36/57 | 38/54 | 64 | 50 | 1.20 (0.76-1.89) | 0.44 |
| Q3 (third quartile) | 21/28 | 53/83 | 63 | 53 | 0.79 (0.47-1.32) | 0.37 |

Table S1 – Overall survival (OS) by CD31 MVD quartile.

Q1: 16.3 mean vessels; Q2: 22 mean vessels; Q3: 29 mean vessels.
