# Supplementary material for: Characterisation of tumour microvessel density during progression of high-grade serous ovarian cancer: clinico-pathological impact (an OCTIPS Consortium study)
Source: Br J Cancer. 2018 Jun 29;119(3):330–8. doi: 10.1038/s41416-018-0157-z (PMC6070919; doi:10.1038/s41416-018-0157-z)
Supplement: Supplementary file 5 — Table S2 (Supplementary) [file 41416_2018_157_MOESM5_ESM.docx]

Table S2 – Correlation between MVD and/or VEGF expression profile in pOC and Tumour Infiltrating Lymphocyte (TILs) phenotype.

| Median number pmf (pOC) |  | MVD^high^ | MVD^low^ | P |  | VEGF^pos^ | VEGF^neg^ | P |  | MVD^high^+VEGF^pos^ | NO  co-expression | P |
| --- | --- | --- | --- | --- | --- | --- | --- | --- | --- | --- | --- | --- |
| **CD3+** |  | **164** | **90** | **0.029** |  | 127 | 159.5 | 0.257 |  | 127 | 158 | 0.432 |
| CD4+ |  | 66 | 113 | 0.151 |  | 71 | 77 | 0.279 |  | 78 | 76 | 0.472 |
| **CD8+** |  | **147.5** | **77** | **0.013** |  | 77 | 125 | 0.204 |  | 94.5 | 122 | 0.364 |
| FoxP3+ |  | 1 | 0.6 | 0.443 |  | 0.4 | 1 | 0.562 |  | 0.6 | 1 | 0.803 |
