# Supplementary material for: Characterisation of tumour microvessel density during progression of high-grade serous ovarian cancer: clinico-pathological impact (an OCTIPS Consortium study)
Source: Br J Cancer. 2018 Jun 29;119(3):330–8. doi: 10.1038/s41416-018-0157-z (PMC6070919; doi:10.1038/s41416-018-0157-z)
Supplement: Supplementary file 6 — Supplementary Figure Captions [file 41416_2018_157_MOESM6_ESM.docx]

**Figure S1 (supplementary):** MVD^high^ and VEGF^(+)^ co-staining frequency among pOCs *versus* rOCs (bar plot). Asterisk indicates significance (p=0.02) between pOCs and rOCs.

**Figure S2 (supplementary):** CD3, CD4, CD8 and FoxP3 staining of intratumoural T lymphocytes.

**Figure S3** **(supplementary):** VEGF-A IRS changes from pOCs to rOCs among BRCA-wt (a - box plot – and c – scatter plot; Wilcoxon test: p=0.126; Spearman correlation test: p=0.290; Spearman coefficient -0.200) *versus* BRCA-mut patients (b – box plot – and d – scatter plot; Wilcoxon test: p=0.053; Spearman correlation test: p=0.226; Spearman coefficient 0.276).
